# Concentration levels of selected essential and toxic metals in potato (*Solanum tuberosum* L.) of West Gojjam, Amhara Region, Ethiopia

**DOI:** 10.1186/s40064-015-1301-3

**Published:** 2015-09-17

**Authors:** Berhe Tadesse, Minaleshewa Atlabachew, Kebede Nigussie Mekonnen

**Affiliations:** Department of Chemistry, College of Science, Bahir Dar University, P. O. Box 079, Bahir Dar, Ethiopia; Department of Chemistry, College of Natural and Computational Science, Mekelle University, P. O. Box 231, Mekelle, Ethiopia

**Keywords:** Potato, *Solanum tuberosum* L., Flame atomic absorption spectrometry, Mineral, Ethiopia

## Abstract

Potato (*Solanum tuberosum* L.) is one of the most widely used as a staple food crop for human diets. It is an excellent source of minerals. In this study, contents of Ca, Mg, Fe, Zn, Cd and Pb in potato cultivars cultivated in Yilmana Densa, and Mecha districts of the West Gojjam zone, Ethiopia were determined by flame atomic absorption spectrometry. A 0.50 g oven-dried potato sample was digested using a mixture of 10 mL HNO_3_:HClO_4_ (4:1 v/v) at 120 °C for 3 h. The concentration ranges in dry weight basis in decreasing order were: Mg (420–438 mg/kg) > Ca (176–254 mg/kg) > Fe (27.3–90.4 mg/kg) > Zn (20.6–77.7 mg/kg) > (2.00–17.4 mg/kg) for Pb. The toxic heavy metal Cd was below the limit of detection in all the analyzed samples (<0.1 mg/kg). The Mg found in highest contents while Fe was the most abundant microelement. The Cd was found below the provisional maximum tolerable daily intake of WHO/FAO and European Commission (EC) while Pb was above the limit. A wide range of variations was observed in the metal contents of potato cultivars collected from the two districts. Potato cultivars grown in West Gojam zone of Ethiopian could contribute a substantial amount of Fe and Zn together with the major elements, Ca and Mg to the individual’s daily dietary needs if consumed on a regular basis.

## Background

Potatoes are tubers belonging to the Solanaceae family (Luis et al. 2011; Nigussie et al. 2014). Research indicated that approximately 5000 varieties of potatoes are available all over the world of which *Solanum tuberosum*, is the most cultivated species (Burlingame et al. 2009; Lutaladio and Castaldi 2009). Potatoes are diverse in tuber shape, size, colour, flavour, taste, texture, storage quality and cooking quality (Luis et al. 2011; Evers and Deußer 2012). Potato has a more dominant place in the diets of people in developed and developing countries since it can grow quickly, cheaply, and freed entire populations from hunger (Burlingame et al. 2009; Lutaladio and Castaldi 2009).

Worldwide currently potato is the fourth most important food crop in production after maize, wheat and rice (FAO 2008, 2009; Ayalew 2014). Furthermore, it is a high-potential food security crop because of its ability to provide high yield of high-quality product per unit input than the major cereal crops like maize (Hirpa et al. 2010). In many of the countries, the poorest and most undernourished farm households depend on potato as a primary or secondary source of nutrition because it produces large quantities of dietary energy and stable yields under conditions in which other crops might fail (Lutaladio and Castaldi 2009).

In addition to its low fat content, potato supplies dietary fibre, carbohydrates, high-quality proteins, vitamins and minerals (Burlingame et al. 2009; Lutaladio and Castaldi 2009). It is a source of antioxidant compounds, including polyphenols, carotenoids and vitamins (Evers and Deußer 2012). The moisture content of freshly harvested potato is about 80 %, where 60–80 % of the remaining dry matter is starch (Lutaladio and Castaldi 2009). The skins and/or fleshes of the ordinarily cultivated varieties of potato are white, yellow or red, which originates from the accumulation of anthocynanis (Zhao et al. 2009). At present, coloured potatoes have attracted special interests in many countries due to their colourful appeals and excellent tastes (Zhao et al. 2009).

Ethiopia is among the top potato producers in Africa, with 70 % of its arable land in the high altitude areas being suitable for potato production (FAOSTAT 2008). In 2013, Ethiopia stood in the 10th position from African countries in the production estimating that production has increased from 280,000 tonnes in 1993 to around 775,503 tonnes in 2013 (FAOSTAT (Food and Agriculture Organization of the United Nations Statistics Division) 2015). Currently, potato is produced mainly in the north western, central, eastern and southern highlands of Ethiopia (CSA 2008/2009; Bekele et al. 2011; Nigussie et al. 2014). The north western part of the country is one of the major production areas and makes up over one third of the total area allotted to potato nationally (CSA 2008/2009; Bekele et al. 2011).

All edible plants are sources of minerals in the diet and also sources of heave metal intoxication to consumers (Islam et al. 2007). Anthropogenic activities, such as mining, industrial and domestic wastewater and sludge, fertilizer and pesticide application to land, as well as atmospheric deposition are the main sources of metal contamination in plants (Szynkowska et al. 2009; Wuana and Okieimen 2011). Among inorganic contaminants, heavy metals are important due to their non-degradable nature leading to bioaccumulation through tropic levels, which may have adverse biological effects (Wagesho 2015). Even at low concentrations, elements such as Ni, Cd, Cr and Pb are harmful to plants and humans (Golia et al. 2008; Kirkillis et al. 2012; Parsafar and Marofi 2014). Potato accumulates the major, minor and toxic elements such as Cd and Pb, which are toxic to humans (Srek et al. 2012). Indeed, the mineral distribution may vary within the potato tuber and geographical location (LeRiche et al. 2009; White et al. 2009; Luis et al. 2011; Subramanian et al. 2011).

Various countries of the world reported the mineral contents of potato cultivars (Navarre et al. 2009; Angelova et al. 2010; Luis et al. 2011; Ozturk et al. 2011; Srek et al. 2012). These findings showed that physico-chemical nature of the soil, geographical locations, agricultural practices and climatic conditions of the various regions had significant influence on the levels of minerals in potato. However, there is a scarcity of information regarding the level of minerals in potato cultivars grown in Ethiopia except few reports on other aspects of potato like blooming its production, paste and disease control mechanisms (Hirpa et al. 2010; Ayalew and Beyene 2011; Mekonen et al. 2011; Ayalew and Beyene 2012; Nigussie et al. 2014). Ethiopia has a unique topography and climatic conditions compared to other part of the world. Therefore, the objective of this study was to determine the concentration level of some selected elements (Ca, Mg, Fe, Zn, Cd and Pb) in potato tubers grown in two selected districts of West Gojam Zone, Amhara Region, Ethiopia.

## Results and discussion

The precision of the results were evaluated by standard deviation and percentage of relative standard deviation (%RSD) of the analyte. The %RSD of the results was less 10 % except for Pb. The limit of detection for each metal was estimated by digesting of the analytical blanks with the optimized procedure of potato samples and calculated by the standard deviation of blank reagents. The moisture content (in %) was determined using the equation:

$${\text{\% moisture}} = \frac{{{\text{Initial weight}} - {\text{Final weight }}}}{\text{Initial weight}} { \times } 100$$

The moisture content of the samples was found to be between 74 and 81 %, which is similar to Ritter et al. (2008) (72–87 %). Similarly Burlingame et al. (2009) reported a range of 63–87 % of water content, which is comparable to the current study. The water content of fresh weight potatoes varies for a number of reasons, variety being one. It is likely that some of the differences in nutrient composition are related to the differences in water content (Burlingame et al. 2009).

## Level of major and minor metals in potato samples

Quantification of minerals in potatoes grown in Ethiopia is relevant because various factors are responsible to affect their concentration in the plant. These facts may include soil pH, cation exchange capacity, organic matter content, types and varieties of plants, fertilizers, pesticides and agricultural practice (Abbas et al. 2012). The concentrations of the selected elements in the studied potato samples followed the trend Mg > Ca > Fe > Zn > Pb > Cd (Table 1). Similar results were also reported elsewhere (Ozturk et al. 2011; Stasinos et al. 2014). Among the analysed metals Cd was below the limit of detection (0.1 mg/kg), while Mg is found in high amount followed by Ca whereas Fe was also found to be high among the tested micronutrient heavy metals followed by Zn.

**Table 1:** Mean elemental contents (mean ± SD, n = 3 mg/kg dry weight) in potato tuber samples collected from Yilmana Densa and Mecha districts

Metals	Mecha		Yilmana Densa	
	Site 1	Site 2	Site 3	Site 4
Ca	176 ± 4	181 ± 5	249 ± 6	254 ± 2
Mg	420 ± 4	426 ± 10	436 ± 7	438 ± 7
Fe	27.3 ± 1.1	40.7 ± 3.4	38.9 ± 0.4	90.4 ± 2.8
Zn	20.6 ± 1.2	28.0 ± 2.4	42.9 ± 4.2	77.7 ± 1.3
Pb	2.0 ± 0.2	6.2 ± 0.7	15.3 ± 0.9	17.4 ± 1.7
Cd	<0.1	<0.1	<0.1	<0.1

*SD* standard deviation; *n* number of measurements

All the studied potato samples were found to contain high amount of Mg (420–438 mg/kg) followed by Ca (176–254 mg/kg). The higher level of Mg in the potato is probably due to the fact that nutrient elements such as N, P, K, S and Mg are found in high amount in the soil. They are easy soluble and highly mobile into the plant tissue (Atlabachew and Chandravanshi 2008). Furthermore, the farmers usually use manure and organic residues as fertilizer to enhance the production. From the studied trace metals, Fe was found to be at higher concentration (27.3–90.4 mg/kg) followed by Zn (20.6–77.7 mg/kg). The higher concentration of these nutrients in the samples could be attributed to their availability in the soil of the farmland. The high concentration of Zn in the studied samples could be due to the usage of organic and phosphate fertilizer as well as fungicides likes mancosim to flourish their production (Dvorak et al. 2003). In this study, the Pb concentration ranged between (2.0–17.4 mg/kg).

Comparing the level of these minerals in the studied samples, wider variations have been noted with respect to the sampling sites. This might be explained by the variations of the aforementioned parameters. When pair-wise comparison was made, a significantly higher concentration of Pb (*P* < *0.05*) was found in the samples obtained from Yilmana Densa district. Similarly, the concentrations of Ca, Fe and Zn were significantly higher (*P* < *0.05*) in samples collected from Yilmana Densa district, where as no significant difference in Mg concentration was noticed when pair-wise analysis done. This study revealed that potato cultivars grown in West Gojam zone of Ethiopian could contribute substantial amount of Fe and Zn together with the major elements, Ca and Mg to the diet if it is consumed on a regular basis. It was reported that deficiencies of micronutrients were a serious problem in Ethiopia (Prinzo and de Benoist 2002; GFDRE-RMH 2011; Herrador et al. 2014). Thus, consumption of potato may alleviate such problem to some extent.

The comparative study of the metal concentration of potato determined in this study and reported values of other researchers are presented in Table 2. The result obtained for Mg was higher than reported in Spain (Luis et al. 2011) and USA (Navarre et al. 2009). The concentration Ca was higher than reported in Spain (Luis et al. 2011) but lower than reported in USA (Navarre et al. 2009). The iron concentration reported in this study was higher than reported in Spain (Luis et al. 2011) and USA (Navarre et al. 2009) but comparable with that of Turkey (Ozturk et al. 2011). The mean concentration of Zn in the presented study was higher than the concentration reported in Spain (Luis et al. 2011) and Turkey (Ozturk et al. 2011) while lower than the one reported in Australia (Angelova et al. 2010). The level of Pb obtained in this study higher than reported in Turkey (Ozturk et al. 2011) but much lower than reported in Australia (Angelova et al. 2010). The level of Cd was much lower than reported in Turkey (Ozturk et al. 2011) and Australia (Angelova et al. 2010). According to the European Commission (EC) and, World Health Organization’s and Food and Agriculture Organization’s (WHO/FAO) permissible limits of heavy metal in foodstuffs, the average concentration of Cd was below the pemissible limit (0.1 mg/kg in potato) while Pb was above the limit (0.1 mk/kg for peeled potato) (EC 2006; WHO/FAO 2011).

**Table 2:** Comparison of determined metals concentration (mg/kg) and dry mass basis with reported values

Metals	Concentration of metal in mg/kg reported in respective country				
	Spain^a^	USA^b^	Turkey^c^	Australia^d^	Ethiopia^e^
Mg	117–361	142–359	–	–	420–438
Ca	13.2–127	455–1300	–	–	176–254
Fe	7.3–14.1	3.0–23.0	48.9–72.6	–	27.3–90.4
Zn	3.0–4.9	–	13.8–18.9	90–110	20.6–77.7
Pb	–	–	0.51–0.77	50.0–54.0	2.0–17.4
Cd	–	–	0.08–0.32	0.40–1.0	<0.1

^a^Luis et al. (2011)

^b^Navarre et al. (2009)

^c^Ozturk et al. (2011)

^d^Angelova et al. (2010)

^e^This study

The results of this study was also compared with data reported by Aregahegn et al. (2013) on other tuber called “Yam” (*Dioscorea abyssinica*) grown in the southern part of Ethiopia. Similar trend in the distribution of the studied metals in the two studies were observed but a significant variation in the range of metals concentration in the two species were seen. Thus, this difference could be explained by the variation due to geographical origin, inter-population and species variability.

## Conclusion

The concentration levels of selected essential and toxic elements in the potato samples followed the trend Mg > Ca > Fe > Zn > Pb > Cd. Potato and *D. abyssinica*, showed similar trends in the distribution of the elemental concentration with a significant variation in the range. The potato tuber contains substantial amounts of Fe and Zn consumed together with the major elements, Ca and Mg on a regular basis. The findings of this study have potential to promote the production and diversification of potato consumption in Ethiopia.

## Methods

### Study area

West Gojjam is a Zone in the Amhara Region of Ethiopia. Mecha and Yilmana Densa are the districts in the West Gojiam where both shared borderlines. Merawi is a town found in Mecha district lying on latitude 11°24′N and longitude of 37°9′E. Adet is a town in Yilmana Densa district with latitude 11°16′N and longitude of 37°29′E. These districts are well known in producing high yield of potato throughout the year via irrigation with the use of agrochemicals. They are identified as a potential for potato production by Amhara Region Agricultural Research Institution (ARARI), Bahir Dar, Ethiopia as the main potato suppliers to Bahir Dar city and other nearby towns.

### Chemicals, reagents and instrumentation

For digestion of potato samples HNO_3_ (69 %) and HClO_4_ (70 %) were used. La(NO_3_) × H_2_O was used to avoid refractory interference. Double distilled water was used for dilution of sample and preparing working standard. All glassware and apparatus were soaked in detergents for 24 h and rinsed with double distilled water. Then they were soaked with 10 % HNO_3_ for 24 h, rinsed with double distilled water and oven-dried at 110 °C. The standard solutions of analytes used for calibration were produced by diluting a stock solution of 1000 mg/L of the given element. Determinations of the selected elements in potato samples were performed using a flame atomic absorption spectrometer (novAA 300, Norwalk, USA) equipped with hallow cathode lamps and working with air-acetylene flame.

### Sample collection, preparation and analysis

Fresh raw potato samples were purchased from the local markets of both towns and kept in plastic bags. In the laboratory, the samples were washed with tap water to remove the adsorbed soil particulates and rinsed with distilled water. The samples were cut into small pieces before being oven dried to constant weight. The samples were then pulverized with a ceramic mortar and pestle to fine powder. About 0.50 g of dried powdered samples were weighed and transferred into Pyrex beaker and were subjected to wet digestion with 10 mL HNO_3_:HClO_4_ (4:1 v/v) at 120 °C for 3 h. Digested samples were allowed to cool, filtered through Whatman^®^ No. 42 filter paper, transferred into 50-mL volumetric flask and diluted to the mark. For each of the samples, triplicate digestions were carried out together with blank reagent and kept in refrigerator until analysis. The samples were analyzed by flame atomic absorption spectrometer (FAAS). The various parameters for maximum signal intensity of the instrument were optimized and analysis was done using external calibration curve with a correlation coefficient ≥0.998 (Table 3). For the analysis of Ca and Mg the working standards were prepared in the presence of 2 mL of La(NO_3_) × H_2_O.

**Table 3:** The FAAS instrument operation condition for determination of selected elements in potato samples

Metals	Wavelength (nm)	Limit of detection (mg/kg)	Slit width (nm)	Lamp current (mA)	Correlation coefficient of calibration curve
Ca	422.7	8.6	1.2	12	0.99997
Mg	285.2	0.5	1.2	6	0.99890
Fe	248.3	5.0	0.2	15	0.99989
Zn	213.9	1.8	0.5	6	0.99988
Pb	283.3	0.3	1.2	6	0.99806
Cd	228.8	0.1	1.2	6	0.99972

### Statistical analysis

Analyses of variance (ANOVA) were computed for statistically significant differences determined by the appropriate F-tests. The results were presented as the mean ± SD for triplicate analysis. Mean differences were compared using the Duncan multiple test (P < 0.05).

## References

[CR1] Abbas G, Hafiz IA, Abbasi NA, Hussain A (2012). Determination of processing and nutritional quality attributes of potato genotypes in Pakistan. Pak J Bot.

[CR3] Angelova V, Ivanova R, Pevicharova G, Ivanov K (2010) Effect of organic amendments on heavy metals uptake by potato plants Australia. Paper presented at 19th World Congress of Soil Science, Soil Solutions for a Changing World, Brisbane, Australia, 1–6 August 2010, pp 84–87

[CR4] Aregahegn A, Chandravanshi BS, Atlabachew M (2013). Levels of major, minor and toxic metals in tubers and flour of Dioscorea abyssinica grown in Ethiopia. Afr J Food Agric Nutr Dev.

[CR5] Atlabachew M, Chandravanshi BS (2008). Levels of major, minor and trace elements in commercially available enset (*Ensete ventricosum* (Welw.), Cheesman) food products (Kocho and Bulla) in Ethiopia. J Food Compost Anal.

[CR6] Ayalew T (2014). Analysis of seed potato (*Solanum tuberosum* L.) systems with special focus in Ethiopia: review. Asian J Agri Res.

[CR7] Ayalew A, Beyene S (2011). The influence of potassium fertilizer on the production of potato (*Solanum tuberosum* L.) at Kembata in southern Ethiopia. J Biol Agri Healthcare.

[CR8] Ayalew A, Beyene S (2012). Characterization of soils and response of potato (*Solanum tuberosum* L.) to application of potassium at Angacha in southern Ethiopia. Int Res J Biochem Bioinform.

[CR9] Bekele B, Abate E, Asefa A, Dickinson M (2011). Incidence of potato viruses and bacterial wilt disease in the west Amhara sub-region of Ethiopia. J Plant Pathol.

[CR10] Burlingame B, Mouille B, Charrondiere R (2009). Nutrients, bioactive non-nutrients and anti-nutrients in potatoes. J Food Comp Anal.

[CR11] Commission Regulation (EC) (2006) No 1881/2006 of 19 December 2006 setting maximum levels for certain contaminants in foodstuffs. Off J Eur Union L 364:5–24

[CR12] CSA (Central Statistical Agency of Ethiopia) (2008/2009) Agricultural sample survey: report on area and production of crops, Addis Ababa, p 126

[CR13] Dvorak P, Tlustos P, Szakova J, Cerny J, Balik J (2003). Distribution of soil fraction of zinc and its uptake by potatoes, maize, wheat and barley after soil amendment by sludge and inorganic zinc salt. Plant Soil Environ.

[CR15] Evers D, Deußer H (2012) Potato antioxidant compounds: impact of cultivation methods and relevance for diet and health, nutrition, well-being and health. In: Dr. Jaouad Bouayed (ed), ISBN: 978-953-51-0125-3, InTech. http://www.intechopen.com/books/nutrition-well-being-and-health/potatoantioxidant-compounds-impact-of-cultivation-methods-and-relevance-for-diet-and-health. Accessed 25 April 2015

[CR16] FAO (Food and Agriculture Organization of the United Nations Rome) (2008) International year of the potato 2008 Food and Agriculture Organization of the United Nations Rome, 2008 New light on a hidden treasure. An end-of-year review

[CR17] FAO (Food and Agriculture Organization of the United Nations) (2009) In: FAOSTAT, 10-5-2011. http://faostat.fao.org. Accessed 25 April 2015

[CR18] FAOSTAT (Food and Agriculture Organization of the United Nations Statistics Division) (2008) Potato world: production and consumption. International year of the potato 2008

[CR19] FAOSTAT (Food and Agriculture Organization of the United Nations Statistics Division) (2015). http://faostat3.fao.org/download/q/qc/e. Accessed 25 April 2015

[CR20] GFDRE-RMH (Government of the Federal Democratic Republic of Ethiopia-Federal Ministry of Health) (2011). Assessment of feasibility and potential benefits of food fortification in Ethiopia.

[CR21] Golia EE, Dimirkou A, Mitsios IK (2008). Influence of some soil parameters on heavy metals accumulation by vegetables grown in agricultural soils of different soil orders. Bull Environ Contam Toxicol.

[CR22] Herrador Z, Sordo L, Gadisa E, Buno A, Gomez-Rioja R, Iturzaeta JM, de Armas LF, Benito A, Aseffa A, Moreno J, Canavate C, Custodio E (2014). Micronutrient deficiencies and related factors in school-aged children in Ethiopia: a cross-sectional study in Libo Kemkem and Fogera Districts, Amhara Regional State. PLoS One.

[CR23] Hirpa A, Meuwissen MPM, Tesfaye A, Lommen WJM, Lansink AO, Tsegaye A, Struik PC (2010). Analysis of seed potato systems in Ethiopia. Am J Pot Res.

[CR24] Kirkillis CG, Pasias IN, Miniadis-Meimaroglou S, Nikolaos ST, Zabetakis I (2012). Concentration levels of trace elements in carrots, onions, and potatoes cultivated in Asopos Region, Central Greece. Anal Lett.

[CR25] LeRiche EL, Wang-Pruski G, Zheljazkov VD (2009). Distribution of elements in potato (*Solanum tuberosum* L.) tubers and their relationship to after-cooking darkening. HortScience.

[CR26] Luis S, Rubio C, Gonzalez-Weller D, Gutierrez AJ, Revert C, Hardisson A (2011). Comparative study of the mineral composition of several varieties of potatoes (*Solanum tuberosum* L.) from different countries cultivated in Canary Islands (Spain). Int J Food Sci Technol.

[CR27] Lutaladio N, Castaldi L (2009). Potato: the hidden treasure. J Food Comp Anal.

[CR28] Mekonen S, Alemu T, Kassa B, Forbes G (2011). Evaluation of contact fungicide spray regimes for control of late blight (*Phytophthora infestans*) in southern Ethiopia using potato cultivars with different levels of host resistance. Trop Plant Pathol.

[CR29] Navarre DA, Goyer A, Shakya R, Singh J, Kaur L (2009). Nutritional value of potatoes. Vitamin, phytonutrient and mineral content. Advances in potato chemistry and technology.

[CR30] Nigussie Z, Alemayehu G, Degefa T, Ngetich K, Tewodros Y, Freyer B (2014). Nature of local seed potato system in northwestern Ethiopia. Int J Agri Res.

[CR31] Ozturk E, Atsan E, Polat T, Kara K (2011). Variation in heavy metal concentrations of potato (*Solanum tuberosum* L.) cultivars. J Anim Plant Sci.

[CR32] Parsafar N, Marofi S (2014). Heavy metal concentration in potato and in the soil via drainage water irrigated with wastewater. Irrig Drain.

[CR33] Prinzo ZW, de Benoist B (2002). Meeting the challenges of micronutrient deficiencies in emergency-affected populations. Proc Nutr Soc.

[CR34] Ritter E, Barandalla L, Lopez R, de Galarreta JIR (2008). Exploitation of exotic, cultivated Solanum germplasm for breeding and commercial purposes. Potato Res.

[CR35] Srek P, Hejcman M, Kunzova E (2012). Effect of long-term cattle slurry and mineral N, P and K application on concentrations of N, P, K, Ca, Mg, As, Cd, Cr, Cu, Mn, Ni, Pb and Zn in peeled potato tubers and peels. Plant Soil Environ.

[CR36] Stasinos S, Nasopoulou C, Tsikrika C, Zabetakis I (2014). The bioaccumulation and physiological effects of heavy metals in carrots, onions, and potatoes and dietary implications for Cr and Ni: a Review. J Food Sci.

[CR37] Subramanian NK, White PJ, Broadley MR, Ramsay G (2011). The three-dimensional distribution of minerals in potato tubers. Ann Bot.

[CR38] Szynkowska MI, Pawlaczyk A, Lesniewska E, Paryjczak T (2009). Toxic metal distribution in rural and urban soil samples affected by industry and traffic. Polish J Environ Stud.

[CR39] ul Islam E, Yang X, He Z, Mahmood Q (2007). Assessing potential dietary toxicity of heavy metals in selected vegetables and food crops. J Zhejiang Univ Sci B.

[CR40] Wagesho Y, Chandravanshi BS (2015). Levels of essential and non-essential metals in ginger (*Zingiber officinale*) cultivated in Ethiopia. SpringerPlus.

[CR41] White PJ, Bradshaw JE, Dale MFB, Ramsay G, Hammond JP, Broadley MR (2009). Relationships between yield and mineral concentrations in potato tubers. HortScience.

[CR42] WHO/FAO (2011) Joint FAO/WHO Food Standards Programme Codex Committee on Contaminants in Foods 5th Session. Working document for information and use in discussions related to contaminants and toxins in the GSCTFF. The Hague, The Netherlands. CF/5 INF/1

[CR43] Wuana RA, Okieimen FE (2011) Heavy metals in contaminated soils: a review of sources, chemistry, risks and best available strategies for remediation. ISRN Eco 2011:20, Article ID 402647. doi:10.5402/2011/402647

[CR44] Zhao CL, Guo HC, Dong ZY, Zhao Q (2009). Pharmacological and nutritional activities of potato anthocyanins. Afri J Pharm Pharmacol.

